# Antihypertensive effects and safety of esaxerenone in patients with moderate kidney dysfunction

**DOI:** 10.1038/s41440-020-00585-y

**Published:** 2020-12-16

**Authors:** Sadayoshi Ito, Hiroshi Itoh, Hiromi Rakugi, Yasuyuki Okuda, Setsuko Iijima

**Affiliations:** 1 Division of Nephrology, Endocrinology and Vascular Medicine, Department of Medicine, Tohoku University School of Medicine, Sendai, Japan; 2 Katta General Hospital, Shiroishi, Japan; 3 Division of Nephrology, Endocrinology and Metabolism, Keio University School of Medicine, Tokyo, Japan; 4 Department of Geriatric and General Medicine, Osaka University Graduate School of Medicine, Suita, Japan; 5 Daiichi Sankyo Co., Ltd., Tokyo, Japan

**Keywords:** Esaxerenone, Japanese, Hypertension, Moderate kidney dysfunction, RAS inhibitor

## Abstract

Renin–angiotensin system inhibitors are recommended for treating hypertension with chronic kidney disease. The addition of a mineralocorticoid receptor blocker may be one option to achieve target blood pressure. We investigated the efficacy and safety of esaxerenone, a mineralocorticoid receptor blocker, in Japanese hypertensive patients with moderate kidney dysfunction. Two multicenter, open-label, nonrandomized dose escalation studies were conducted to investigate esaxerenone monotherapy and add-on therapy to renin–angiotensin system inhibitor treatment. Esaxerenone therapy was initiated at 1.25 mg/day and titrated to 2.5 and then 5 mg/day for a treatment duration of 12 weeks. Primary endpoints were changes from baseline in sitting systolic and diastolic blood pressure. Safety, pharmacokinetics, and urinary albumin-to-creatinine ratios were also assessed. Thirty-three patients received monotherapy, and 58 received add-on therapy; the mean baseline estimated glomerular filtration rates were 51.9 and 50.9 mL/min/1.73 m^2^, respectively. The esaxerenone dosage was increased to ≥2.5 mg/day in 100% (*n* = 33) and 93.1% (*n* = 54) of patients receiving monotherapy and add-on therapy, respectively. Reductions in sitting blood pressure from baseline to the end of treatment were similar (monotherapy: −18.5/−8.8 mmHg; add-on therapy: −17.8/−8.1 mmHg; both *P* < 0.001). The antihypertensive effects of esaxerenone were consistent across patient subgroups. A serum K^+^ level ≥5.5 mEq/L was observed in seven patients (12.1%) receiving add-on therapy but in none receiving monotherapy. All increases in serum K^+^ levels were transient, and no patient met predefined serum K^+^ level criteria for dose reduction or therapy discontinuation. No patient discontinued treatment owing to kidney function decline. Esaxerenone was effective and well tolerated in hypertensive patients with moderate kidney dysfunction.

## Introduction

Both hypertension and chronic kidney disease (CKD) are important cardiovascular risk factors and form a vicious cycle [1–7]. However, hypertensive patients with CKD often have an inadequate response to antihypertensive drugs. For those with moderate kidney dysfunction, renin–angiotensin system (RAS) inhibitors, including angiotensin receptor blockers (ARBs) and angiotensin-converting enzyme inhibitors (ACEis), are recommended as initial therapies [7]. However, RAS inhibitor monotherapy is often insufficient to achieve blood pressure (BP) control in these patients, and the addition of a mineralocorticoid receptor blocker is one option for CKD patients with treatment-resistant hypertension [7–10]. This is because mineralocorticoid receptor activity is usually enhanced in these patients, even during RAS inhibitor treatment.

Spironolactone and eplerenone are currently available mineralocorticoid receptor blockers [11, 12]. However, sex hormone-related adverse events or hyperkalemia is problematic [13–16], suggesting that careful management is required. In the United States, European Union, and Japan, eplerenone is contraindicated in patients with moderate kidney dysfunction (creatinine clearance <50 mL/min) and in diabetes patients with microalbuminuria or proteinuria [14], limiting its use in these populations.

Esaxerenone is a novel nonsteroidal oral mineralocorticoid receptor blocker that potently and specifically inhibits excessive mineralocorticoid receptor activity [17]. The urinary excretion of unchanged esaxerenone in humans is as low as 1.6% [18], suggesting that plasma exposure to esaxerenone is not affected by kidney dysfunction. This property would make it suitable for CKD patients with or without type 2 diabetes [18]. Preclinical data have shown that esaxerenone suppresses urinary protein excretion and histopathological kidney damage, even at low doses [19–21]. We previously reported the effects of esaxerenone in hypertensive patients with normal kidney function [22, 23] and in diabetic patients with albuminuria [24, 25]. Herein, we report findings from two studies that were carried out in a different patient population represented by hypertensive patients with moderate kidney dysfunction who do not have diabetes with albuminuria to evaluate the efficacy and safety of esaxerenone monotherapy and add-on therapy to a RAS inhibitor.

## Methods

### Study design

Two single-arm, open-label, nonrandomized, dose escalation studies were conducted in Japan: a monotherapy study (NCT02448628) at two centers from May to November 2015 and an add-on therapy study (NCT02807987) at 14 centers from June 2016 to May 2017. The monotherapy study was the first to administer esaxerenone to hypertensive patients with moderate kidney dysfunction. Therefore, at study initiation, all patients were admitted to hospital to ensure their safety, and their pharmacokinetics, BP, K^+^ levels, and estimated glomerular filtration rate (eGFR) were measured. After these parameters were carefully evaluated, the add-on therapy study commenced.

Ethical approval was obtained from all independent institutional review boards, and procedures were performed in accordance with the Declaration of Helsinki and Good Clinical Practice. All patients provided written informed consent.

### Patients

Eligible patients were aged 20–80 years, had an average systolic BP between ≥140 and <180 mmHg based on two measurements in the observational period, an average diastolic BP between ≥80 and <110 mmHg (between-measurement difference ≤30/15 mmHg), and an eGFR between ≥30 and <60 mL/min/1.73 m^2^. In the add-on therapy study, patients were also receiving a RAS inhibitor at a stable dose for ≥4 weeks prior to study drug administration. Patients with a serum K^+^ level <3.5 or ≥5.1 mEq/L were excluded from the monotherapy study, while those with a serum K^+^ level <3.5 or ≥4.8 mEq/L were excluded from the add-on therapy study. Patients were also excluded if they had been diagnosed with malignant or secondary hypertension (except for renal parenchymal hypertension) or if they had diabetes with albuminuria (urine albumin-to-creatinine ratio [UACR] ≥ 30 mg/g∙Cr). Concomitant use of other antihypertensives was prohibited. The eGFR was calculated from serum creatinine using the following equation:

eGFR = 194 × serum creatinine^−1.904^ × age^−0.287^ (multiplied by 0.739 for women).

### Intervention

**Fig. 1:**
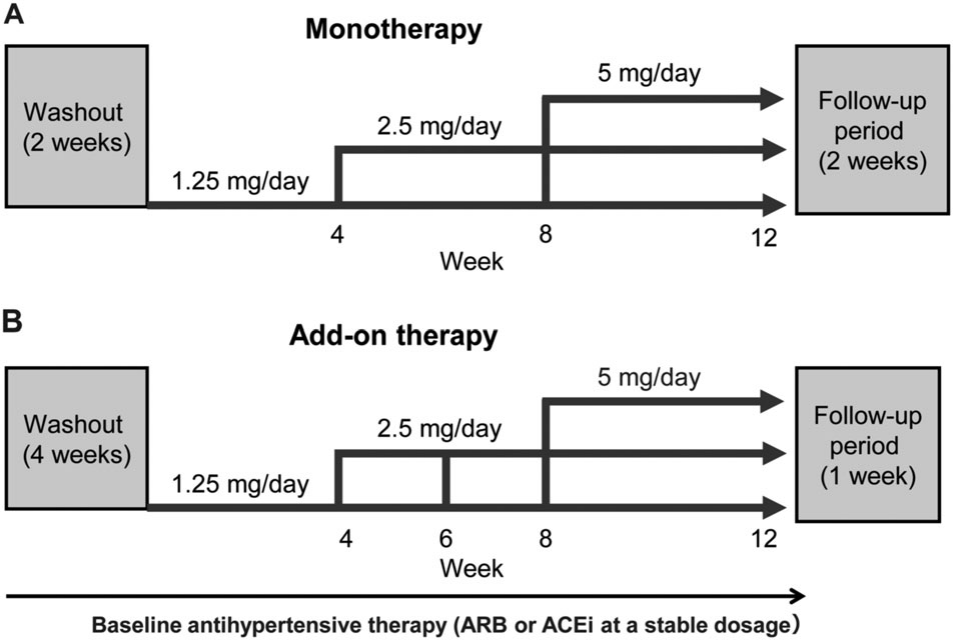
Design of the esaxerenone monotherapy (**a**) and add-on therapy (**b**) studies. ACEi angiotensin-converting enzyme inhibitor, ARB angiotensin receptor blocker

In the monotherapy study, all antihypertensive agents were washed out during a 2-week washout period. In the add-on therapy study, all antihypertensive agents except for the RAS inhibitor were washed out during a 4-week washout period. Oral esaxerenone was started at a dose of 1.25 mg once daily for 4 weeks, followed by dose escalation and follow-up periods (Fig. 1). In the monotherapy study, the esaxerenone dosage was increased from 1.25 to 2.5 mg/day at week 4 or 8 and then from 2.5 to 5 mg/day at week 8. In the add-on therapy study, the esaxerenone dosage was increased from 1.25 to 2.5 mg/day at week 4, 6 or 8. To allow a 4-week observation period at any one dose, the dosage was increased from 2.5 to 5 mg/day at week 8 only in patients whose dose was increased to 2.5 mg at week 4 (Fig. 1).

Dose escalation for both studies was considered when the serum K^+^ level was <5.1 (monotherapy) or <4.8 (add-on therapy) mEq/L and when the eGFR reduction from baseline was <30% based on physician discretion to achieve a BP reduction of <130/80 mmHg.

Esaxerenone was discontinued if the serum K^+^ level was ≥6.0 mEq/L on one occasion or ≥5.5 mEq/L on two consecutive occasions in all monotherapy patients and in add-on therapy patients receiving 1.25 mg/day. For add-on therapy patients receiving 2.5 or 5 mg/day, the dose was reduced if the serum K^+^ level was ≥6.0 or ≥5.5 mEq/L on two consecutive measurements; if the serum K^+^ level remained ≥5.5 mEq/L after dose reduction, treatment was discontinued. Dose reductions were also made if severe hypotension developed or if deemed necessary by the investigator.

### Efficacy assessments

The primary endpoints were changes in sitting systolic/diastolic BP from baseline (average of values measured at the last two visits during the observational period) to the end of treatment (average of values measured at the last two visits during treatment). Secondary efficacy endpoints in both studies included changes over time in sitting systolic/diastolic BP and the proportion of patients achieving target BP (<140/90 mmHg). Additional exploratory endpoints in both studies were changes in the UACR from baseline to week 12. Plasma renin activity (PRA) was measured at baseline and every 4 weeks (monotherapy) or at baseline and week 12 (add-on therapy). Trough plasma esaxerenone concentrations were measured every 2 weeks (monotherapy) or every 4 weeks (add-on therapy). Changes in kidney injury biomarkers (liver-type fatty acid-binding protein [L-FABP], N-acetyl-beta-(D)-glucosaminidase [NAG], β2 microglobulin [β2-MG], angiotensinogen [ATG], and 8-hydroxy-2′-deoxyguanosine [8-OhdG]) were also measured in the add-on therapy study.

BP was measured just before the next esaxerenone dose using the same automated sphygmomanometer (TM-2433; A&D Co., Ltd.); the mean of three measurements was calculated. In the monotherapy study, patients were hospitalized from the day before the date of esaxerenone therapy initiation to the day after initiation. BP was measured just before the first administration, and the trough BP level was determined 24 h after the first dose. The UACR was determined in the first morning urine sample. Urine was collected once for nondiabetic patients or twice for diabetic patients during the observation period and at week 12.

### Safety and pharmacokinetic assessments

All adverse events were assessed; laboratory parameter assessments were performed centrally, but local values were used for dose-escalation decision-making. In addition, electrocardiogram findings, vital signs, and the proportion of patients with serum K^+^ levels ≥6.0 mEq/L or ≥5.5 mEq/L on two consecutive measurements were determined. In the monotherapy study, the serum K^+^ level and eGFR were measured on the day before treatment initiation, 24 h after the first dose, every week thereafter, and at the end of the follow-up period. In the add-on therapy study, the serum K^+^ level and eGFR were measured before, 1 and 2 weeks after treatment initiation, every 2 weeks thereafter, and at the end of the follow-up period. In addition, these parameters were measured 1 week after each dose escalation in individuals whose dose was increased.

### Statistical analyses

Based on feasibility and the ability to clinically assess efficacy and safety endpoints, the planned sample sizes were 30 and 50 patients in the monotherapy and add-on therapy studies, respectively. The predefined full analysis set included patients who had taken ≥1 dose of the study drug and had ≥1 postbaseline measurement available. The safety analysis set included all patients who received ≥1 dose of the study drug.

Regarding the primary endpoints, mean changes from baseline to the end of treatment and 95% confidence intervals (CI) were calculated and assessed using paired *t*-tests. The last observation carried forward method was used to impute missing BP data. The proportion of patients achieving target BP was assessed by the point estimate and corresponding exact 95% CI. For other exploratory endpoints, the geometric mean percent change from baseline and the 95% CI were calculated using log-transformed values and assessed using paired *t*-tests. *Post hoc* analyses of the change in BP over time were performed using paired *t*-tests. All reported *P* values and 95% CIs are two-sided, with a significance level of 5%, and were not adjusted for multiple testing. Analyses were performed using SAS System Version 9.2 or 9.3 (SAS Institute Japan Ltd., Tokyo, Japan).

## Results

### Patient disposition

Thirty-three and 58 patients were enrolled in the esaxerenone monotherapy and add-on therapy studies, respectively. The majority of patients were male (72.7% and 77.6%, respectively) and aged ≥65 years (51.5% and 70.7%, respectively), and the mean eGFRs were 51.9 and 50.9 mL/min/1.73 m^2^, respectively (Table 1). In the add-on therapy study, RAS inhibitor therapy was an ARB in 96.6% of patients. Three patients withdrew from each study; two due to adverse events (altered consciousness, chest pain) and one due to persistent BP elevation in the monotherapy study; and one patient each withdrew due to an adverse event (mild hepatic dysfunction), nonattendance at week 12, and insufficient BP control in the add-on therapy study.

**Table 1:** Baseline patient demographics

	Esaxerenone	
	Monotherapy (*n* = 33)	Add-on therapy (*n* = 58)
Male, *n* (%)	24 (72.7)	45 (77.6)
Age, years	63.9 ± 7.9	68.0 ± 7.7
≥65 years, *n* (%)	17 (51.5)	41 (70.7)
Body mass index, kg/m^2^	25.7 ± 3.1	25.2 ± 3.9
≥25 kg/m^2^, *n* (%)	16 (48.5)	29 (50.0)
Systolic BP, mmHg	153.6 ± 7.3	159.4 ± 10.9
≥160 mmHg, *n* (%)	7 (21.2)	30 (51.7)
Diastolic BP, mmHg	93.4 ± 6.7	91.8 ± 7.3
≥100 mmHg, *n* (%)	6 (18.2)	8 (13.8)
eGFR, mL/min/1.73 m^2^	51.9 ± 7.2	50.9 ± 6.5
<45 mL/min/1.73 m^2^, *n* (%)	7 (21.2)	12 (20.7)
Serum K^+^, mEq/L	4.2 ± 0.3	4.3 ± 0.3
≥4.5 mEq/L, *n* (%)	5 (15.2)	17 (29.3)
Hypertension duration, years	11.4 ± 10.7	11.0 ± 6.6
Pretreatment with antihypertensive agents, *n* (%)	24 (72.7)	51 (87.9)
ARB	–	56 (96.6)
ACEi	–	2 (3.4)
Diabetes, *n* (%)	13 (39.4)	13 (22.4)
HbA1c, %	6.1 ± 0.8	5.8 ± 0.5
≥6.9%, *n* (%)	7 (21.2)	2 (3.4)
Blood glucose, mg/dL	115.6 ± 20.5	104.2 ± 17.7
UACR, mg/g•Cr	16.7 ± 33.5	49.5 ± 112.1^a^
≥30 mg/g•Cr, *n* (%)	3 (9.1)	13 (22.4)^a^
Plasma aldosterone concentration, pg/mL	108.1 ± 45.0	93.9 ± 34.7
Plasma renin activity, ng/mL/h	0.8 ± 0.7	1.6 ± 1.6^b^

Values are means ± SDs or numbers of patients (%)

*ACEi* angiotensin-converting enzyme inhibitor, *ARB* angiotensin II receptor blocker, *BP* blood pressure, *eGFR* estimated glomerular filtration rate, *HbA1c* glycated hemoglobin, *UACR* urine albumin-to-creatine ratio

^a^*n* = 57

^b^*n* = 55

Esaxerenone was increased to 2.5 mg/day in all patients in the monotherapy study and in 54/58 patients (93.1%) in the add-on therapy study, and to 5 mg/day in 30/33 patients (90.9%) in the monotherapy study and 25/58 patients (43.1%) in the add-on therapy study. No patients in either study had their esaxerenone dose reduced. In all patients in the monotherapy group who completed the study (*n* = 30), the esaxerenone dosage was increased to 5 mg/day. In the add-on therapy group (*n* = 55), 4, 26 and 25 patients completed the study at dosages of 1.25, 2.5 and 5 mg/day, respectively (Supplementary Fig. 1). Reasons for no dose escalation from 1.25 to 2.5 mg/day in four patients were not required due to adequate BP control (*n* = 3) or not meeting the dose escalation criteria for serum K^+^ levels (*n* = 1). Reasons for not increasing the dose from 2.5 mg to 5 mg in 13 patients were as follows: not meeting the K^+^ criteria (*n* = 5), adequate BP control (*n* = 1), meeting both the K^+^ and BP control criteria (*n* = 1), and the doctor’s discretion (*n* = 6).

### Efficacy

**Fig. 2:**
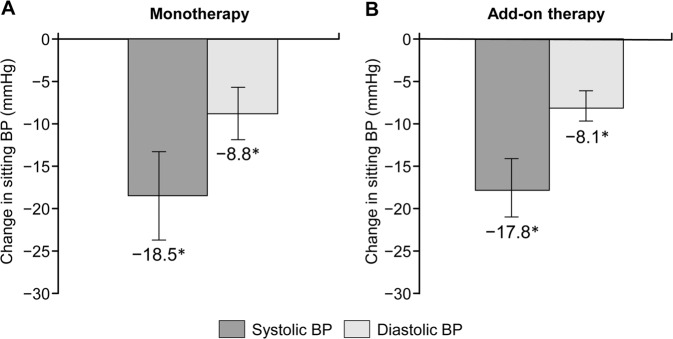
Change in sitting blood pressure (BP) from baseline to the end of treatment for esaxerenone monotherapy (**a**) and add-on therapy (**b**) (full analysis set). Values are shown as the means with 95% confidence intervals. *P < 0.001 for change from baseline (paired *t*-test; last observation carried forward method)

Mean changes in sitting systolic/diastolic BP from baseline to the end of treatment were −18.5 (95% CI −23.7, −13.3)/−8.8 mmHg (95% CI −11.9, −5.7) (monotherapy; *P* < 0.001) and −17.8 (95% CI −21.0, −14.7)/−8.1 (95% CI −9.7, −6.5) mmHg (add-on therapy; *P* < 0.001) (Fig. 2). BP decreased substantially at the dosage of 1.25 mg/day and decreased further after each esaxerenone dose escalation in both groups (Supplementary Figs 2 and 3). The proportions of patients achieving target BP (<140/90 mmHg) at the end of treatment were 63.6% (95% CI 45.1, 79.6; monotherapy) and 48.3% (95% CI 35.0, 61.8; add-on therapy) (Supplementary Fig. 4). The antihypertensive effects of esaxerenone in both studies were adequate across all the predefined patient subgroups based on age, body mass index, the presence/absence of diabetes, and the eGFR, while numerical differences in age, diabetes, and the eGFR were observed in the monotherapy study (Supplementary Table 1). In the add-on therapy study, BP reduction was not dose-dependent with respect to the final dose of esaxerenone (Supplementary Fig. 5).

### Additional endpoints

The mean UACR decreased significantly from baseline to week 12: by 25.5% (95% CI −37.8, −10.8) in the monotherapy study (*P* < 0.01) and 28.6% (95% CI −40.7, −14.0) in the add-on therapy study (*P* < 0.001) (Supplementary Fig. 6). In the add-on therapy study, the majority of kidney injury biomarkers were unchanged, with the exception of a mean 16.4% (95% CI −30.1, −0.1) decrease in AGT and a 37.0% (95% CI −49.5, −21.3) decrease in β2-MG at week 12 (Supplementary Table 2).

### Pharmacokinetic assessments and PRA

Trough plasma esaxerenone concentrations increased in a dose-dependent manner in both studies regardless of stratification by the baseline eGFR (Supplementary Fig. 7).

Esaxerenone increased PRA during the treatment period, with a significant increase from baseline observed at week 12 for the monotherapy group (geometric mean percent change 155.6%; 95% CI 98.7, 228.6) and the add-on therapy group (geometric mean percent change 53.7%; 95% CI 2.3, 130.8) (Supplementary Fig. 8).

### Safety

In the monotherapy study, 21/33 patients (63.6%) experienced an adverse event (mostly of mild severity); the most common events were nasopharyngitis and arthropod sting (both 9.1%) (Table 2). Drug-related adverse events occurred in 8/33 patients (24.2%) (Table 2). No severe adverse events or deaths occurred in either study.

**Table 2:** Summary of adverse events

Adverse events, *n* (%)	Esaxerenone	
	Monotherapy (*n* = 33)	Add-on therapy (*n* = 58)
All adverse events	21 (63.6)	35 (60.3)
Serious adverse events	0 (0.0)	0 (0.0)
Drug-related treatment-emergent adverse events	8 (24.2)	17 (29.3)
Discontinued due to treatment-emergent adverse events^a^	2 (6.1)	1 (1.7)
Adverse events (*n* ≥2 in either study)		
Influenza	–	2 (3.4)
Nasopharyngitis	3 (9.1)	–
Viral upper respiratory tract infection	–	7 (12.1)
Constipation	2 (6.1)	–
Diarrhea	1 (3.0)	2 (3.4)
Dizziness	–	2 (3.4)
Head discomfort	–	2 (3.4)
Blood creatinine increased	–	3 (5.2)
Blood potassium increased	1 (3.0)	6 (10.3)
Blood urea increased	–	2 (3.4)
Blood uric acid increased	2 (6.1)	3 (5.2)
Hematuria	2 (6.1)	–
eGFR decreased	1 (3.0)	3 (5.2)
Arthropod sting	3 (9.1)	–
Change in serum K^+^ level		
Serum K^+^ ≥5.5 mEq/L	0 (0.0)	7 (12.1)
Serum K^+^ ≥6.0 mEq/L or ≥5.5 mEq/L on two consecutive occasions	0 (0.0)	0 (0.0)

*eGFR* estimated glomerular filtration rate

^a^In monotherapy, two patients discontinued due to adverse events, one experienced altered consciousness and the other had chest pain, and the first event was judged to be related to the study drug. In add-on therapy, one patient discontinued due to mild abnormal hepatic function that was judged to be related to the study drug and returned to the normal range after the study drug was discontinued

The overall rate of adverse events in the add-on therapy study was 60.3% (35/58), and drug-related adverse events occurred in 29.3% of patients (17/58) (Table 2).

**Fig. 3:**
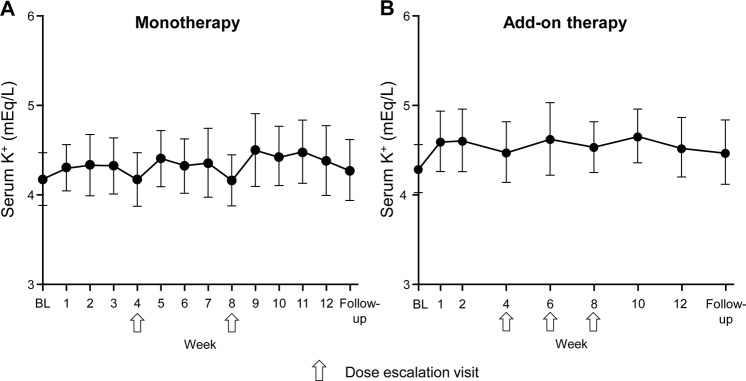
Mean change in serum K^+^ levels over time in the esaxerenone monotherapy (**a**) and add-on therapy (**b**) studies (safety analysis set). Values are shown as the mean ± SD. Arrows indicate dose escalation visits. BL baseline

In both studies, the mean serum K^+^ levels increased after esaxerenone initiation and after each dose escalation visit; the maximum increases from baseline were 0.3 ± 0.3 mEq/L (week 9, *n* = 33) and 0.4 ± 0.3 mEq/L (week 10, *n* = 56) in the monotherapy and add-on therapy groups, respectively. In the add-on therapy group, the largest increase at one week after dose escalation (0.5 ± 0.2 mEq/L, week 7) was observed in patients whose dosage was increased from 1.25 to 2.5 mg/day at week 6 (*n* = 7). There was no trend of a continuous increase in the serum K^+^ level over time (Fig. 3). An increased serum K^+^ level as an adverse event occurred in one and six patients with monotherapy and add-on therapy, respectively. No patient in the monotherapy study and seven patients in the add-on therapy study (two receiving 1.25 mg/day and five receiving 2.5 mg/day) had transient serum K^+^ levels ≥5.5 mEq/L during treatment (Table 2); all completed 12 weeks of therapy without meeting dose reduction or treatment discontinuation criteria.

**Fig. 4:**
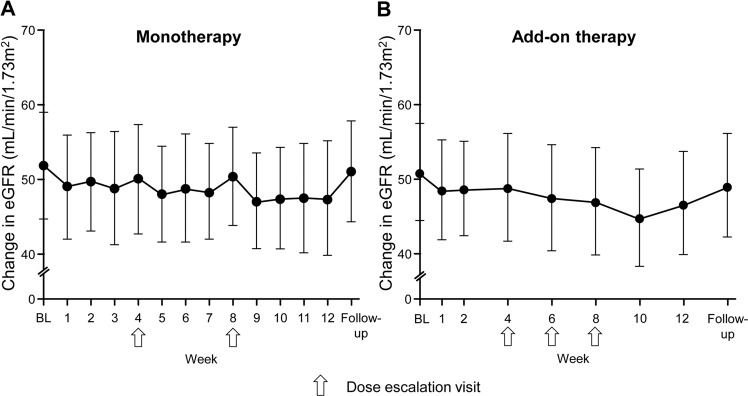
Mean change in the estimated glomerular filtration rate (eGFR) over time in the esaxerenone monotherapy (**a**) and add-on therapy (**b**) studies (safety analysis set). Values are shown as the mean ± SD. Arrows indicate dose escalation visits. BL baseline

In both study groups, the eGFR decreased at week 1 and after each esaxerenone dose escalation (Fig. 4). The eGFR decreased by −4.78 ± 5.15 mL/min/1.73 m^2^ from baseline to week 12 in the monotherapy study and by −4.45 ± 4.44 mL/min/1.73 m^2^ in the add-on therapy study and returned almost to baseline values at the end of the follow-up period in both studies.

Adverse events related to decreased kidney function were an eGFR decrease (*n* = 1) in the monotherapy study and increased serum creatinine (*n* = 3) and an eGFR decrease (*n* = 3) in the add-on therapy study (Table 2). No patient in either study discontinued treatment due to kidney function decline.

The incidence of any adverse event in patients with a baseline eGFR <45 vs. ≥45 mL/min/1.73 m^2^ was similar in the monotherapy (57.1% vs. 65.4%) and add-on (58.3% vs. 60.9%) studies. In the add-on therapy study, a serum K^+^ level increase to ≥5.5 mEq/L was not observed in any patient with an eGFR <45 mL/min/1.73 m^2^ and occurred in 15.2% of patients with an eGFR ≥45 mL/min/1.73 m^2^. Among the adverse events related to decreased kidney function, an increased serum creatinine level and a decreased eGFR occurred in 0.0% and 3.8% (monotherapy), and 25.0% and 6.5% (add-on therapy) of patients with an eGFR <45 mL/min/1.73 m^2^ and ≥45 mL/min/1.73 m^2^, respectively.

### Acute effects of esaxerenone in the monotherapy study

In the monotherapy study, all patients were admitted to hospital to ensure their safety. The mean systolic/diastolic BP decreased from 150.5/91.2 mmHg before therapy initiation to 143.3/88.5 mmHg at 24 h after initial oral administration. The mean serum K^+^ level (±SD) remained unchanged (4.2 ± 0.3 mEq/L before, 4.2 ± 0.3 mEq/L after), and changes in the eGFR were not remarkable (51.9 ± 7.2 mL/min/1.73 m^2^ before, 53.6 ± 7.3 mL/min/1.73 m^2^ after). There were no acute adverse events.

## Discussion

Hypertensive patients with moderate kidney dysfunction are at high cardiovascular risk, necessitating strict BP control. However, the RAS inhibitors recommended for these patients are often insufficient to achieve target BP, and the addition of ≥1 antihypertensive agent is required. We previously evaluated the antihypertensive effect of the novel mineralocorticoid receptor blocker esaxerenone in essential hypertensive patients [22, 23] and in hypertensive patients with diabetes and albuminuria [24, 25]. This report describes findings from two studies in which esaxerenone was administered to patients with moderate kidney dysfunction and without diabetes with albuminuria. We evaluated esaxerenone as a monotherapy or an add-on to RAS inhibitor therapy and demonstrated sufficient antihypertensive effects in both studies. The antihypertensive effects of esaxerenone were −18.5/−8.8 mmHg in the monotherapy group and −17.8/−8.1 mmHg in the add-on therapy group, comparable with that of 5 mg esaxerenone treatment in patients with normal kidney function (−16.9/−8.4 mmHg) in the phase 3 study [22]. PRA was significantly increased throughout treatment with esaxerenone, similar to the effect observed in hypertensive patients with normal kidney function [22]. This suggests effective mineralocorticoid receptor blockade with esaxerenone even in patients with moderate kidney dysfunction.

Notably, the BP-lowering effect of esaxerenone was quite rapid. In the monotherapy study, patients were hospitalized for the initiation of esaxerenone and systolic/diastolic BP decreased from 150.5/91.2 to 143.3/88.5 mmHg 24 h after the first administration; these values were similar to BP values observed after 1 week of treatment.

Previous studies evaluating the antihypertensive effect of mineralocorticoid receptor blockers in patients with moderate kidney dysfunction are limited. Two retrospective studies of a small number of patients with difficult-to-control hypertension or resistant hypertension with stage 3/4 CKD were reported. In these studies, most patients who had received multiple antihypertensive drugs, including ARB and diuretics and add-on therapy with a mineralocorticoid receptor blocker (mostly spironolactone), showed a decrease in systolic BP by 10–24 mmHg and in diastolic BP by 3.4–13 mmHg [26, 27]. Although we cannot make direct comparisons, the antihypertensive effect of esaxerenone appears to be comparable to that of spironolactone.

In the add-on therapy study, 93.1% and 43.1% of patients had their esaxerenone dosage escalated to 2.5 and 5 mg/day, respectively. Reasons for not increasing the dose were a sufficient decrease in BP or not meeting the dose escalation criteria for serum K^+^ levels. The BP-lowering effects of esaxerenone were similar in patients with a final dosage of 1.25, 2.5 or 5 mg/day. This may suggest individual variations in BP responses to esaxerenone. Nevertheless, with close monitoring of patients, the esaxerenone dose can be adjusted to confer substantial BP-lowering benefits in various patient subgroups with moderate kidney dysfunction.

Significant reductions in the UACR were observed even when esaxerenone was added to a RAS inhibitor, consistent with clinical studies on type 2 diabetes that demonstrated a dose-dependent reduction in the UACR [24, 25]. However, the dose dependency of UACR reduction was not statistically significant in the present study because of the low baseline UACR, so its long-term clinical relevance remains unclear in this patient population. Preclinical data show that esaxerenone ameliorates kidney injury independently of its antihypertensive action [19], consistent with other mineralocorticoid receptor blockers [28, 29]. A significant reduction in the urinary biomarker β2-MG (a marker of kidney tubule damage) was also observed with esaxerenone add-on therapy.

Esaxerenone was well tolerated in both studies. Sex hormone-related adverse events, which occur frequently with spironolactone [13, 30, 31], were not observed in either study, consistent with other esaxerenone studies [22, 23]. Hyperkalemia is another safety concern with mineralocorticoid receptor blockers, particularly in the presence of decreased kidney function. In the present study, elevations in the serum K^+^ level were transient, and no patient met dose reduction or therapy discontinuation criteria, even when esaxerenone was added to a RAS inhibitor. In the add-on study, seven of 58 patients had transient serum K^+^ levels ≥5.5 mEq/L, which is comparable to the rate with low-dose spironolactone added to a RAS inhibitor in nondiabetic early-stage CKD patients (nine of 56 patients) [32]. The same study showed that spironolactone decreased the eGFR by 3 mL/min/1.73 m^2^ [32], and another study in nondiabetic CKD patients (baseline eGFR ≤60 mL/min/1.73 m^2^) showed a decrease of 15.1% [33]. In the present study, a similar decrease in the eGFR was observed until week 12 (−4.45 mL/min/1.73 m^2^: −8.56%), but the eGFR returned to baseline levels during the follow-up period. A transient eGFR decline has been reported with all mineralocorticoid receptor blockers, and this decline is thought to be due to the improvement in renal glomerular hyperfiltration [34]. Thus, esaxerenone seems to have a safety profile comparable to that of low-dosage (25 mg/day) spironolactone in patients with moderate kidney dysfunction treated with a RAS inhibitor.

The incidence of adverse events related to kidney function was higher in patients with a baseline eGFR <45 mL/min/1.73 m^2^ than in those with a baseline eGFR ≥45 mL/min/1.73 m^2^, although all patients recovered without study drug discontinuation. Plasma concentrations of esaxerenone were not affected by the baseline eGFR in either study and were consistent with those reported in a phase 3 study of esaxerenone in patients with normal kidney function [22]. Therefore, esaxerenone can be safely administered by starting at a low dosage with careful monitoring, even in those with an eGFR <45 mL/min/1.73 m^2^, especially when esaxerenone is added to a RAS inhibitor.

Starting esaxerenone from a low dosage (1.25 mg/day) and incrementally escalating the dose based on the serum K^+^ level, eGFR, and BP response may have contributed to the good tolerability observed in this study. The esaxerenone dosage was safely escalated to 2.5 mg/day in all patients receiving monotherapy and the majority (93.1%) of patients receiving add-on therapy. Further, the esaxerenone dosage was safely escalated to 5 mg/day in most patients (90.9%) in the monotherapy study and almost half of the patients (43.1%) in the add-on therapy study. In a previous study of eplerenone, dose escalation based on the BP response also appeared to minimize the risk of hyperkalemia compared with forced escalation [35].

The limitations of the present study were its nonrandomized design, small sample sizes, and relatively short study period. Furthermore, this study included only Japanese patients, so generalizability to other patient populations may be limited.

In conclusion, esaxerenone demonstrated sufficient antihypertensive effects and was well tolerated in hypertensive patients with moderate kidney dysfunction, both as monotherapy and add-on therapy to a RAS inhibitor. The magnitude of antihypertensive effects was comparable in hypertensive patients with normal kidney function. Serum K^+^ elevation was manageable by titrating from a low starting dose.

## Supplementary information

Supplementary Information
