# Supplementary material for: Antihypertensive effects and safety of esaxerenone in patients with moderate kidney dysfunction
Source: Hypertens Res. 2020 Dec 16;44(5):489–97. doi: 10.1038/s41440-020-00585-y (PMC8099724; doi:10.1038/s41440-020-00585-y)
Supplement: Supplementary file 1 — Supplementary Information [file 41440_2020_585_MOESM1_ESM.docx]

# Antihypertensive effects and safety of esaxerenone in patients with moderate kidney dysfunction

**Running title:** Esaxerenone in hypertensives with moderate kidney dysfunction

Sadayoshi Ito^1,2^, Hiromi Rakugi^3^, Hiroshi Itoh^4^, Yasuyuki Okuda^5^, Setsuko Iijima^5^

^1^Division of Nephrology, Endocrinology and Vascular Medicine, Department of Medicine, Tohoku University School of Medicine, Sendai, Japan

^2^Katta General Hospital, Shiroishi, Japan, Sendai, Japan

^3^Department of Geriatric and General Medicine, Osaka University Graduate School of Medicine, Suita, Japan

^4^Division of Nephrology, Endocrinology and Metabolism, Keio University School of Medicine, Tokyo, Japan

^5^Daiichi Sankyo Co., Ltd., Tokyo, Japan^*^Katta General Hospital, Shiroishi, Japan (current affiliation)

**Corresponding author:**

Sadayoshi Ito, MD, PhD

Division of Nephrology, Endocrinology and Vascular Medicine, Department of Medicine, Tohoku University School of Medicine, 2-1 Seiryo-machi, Aoba, Sendai, Miyagi 980-8575, Japan

Tel: +81-22-717-7163

Fax: +81-22-717-7168

E-mail: db554@med.tohoku.ac.jp

# Supplementary Figures

**Supplementary Figure 1**

**
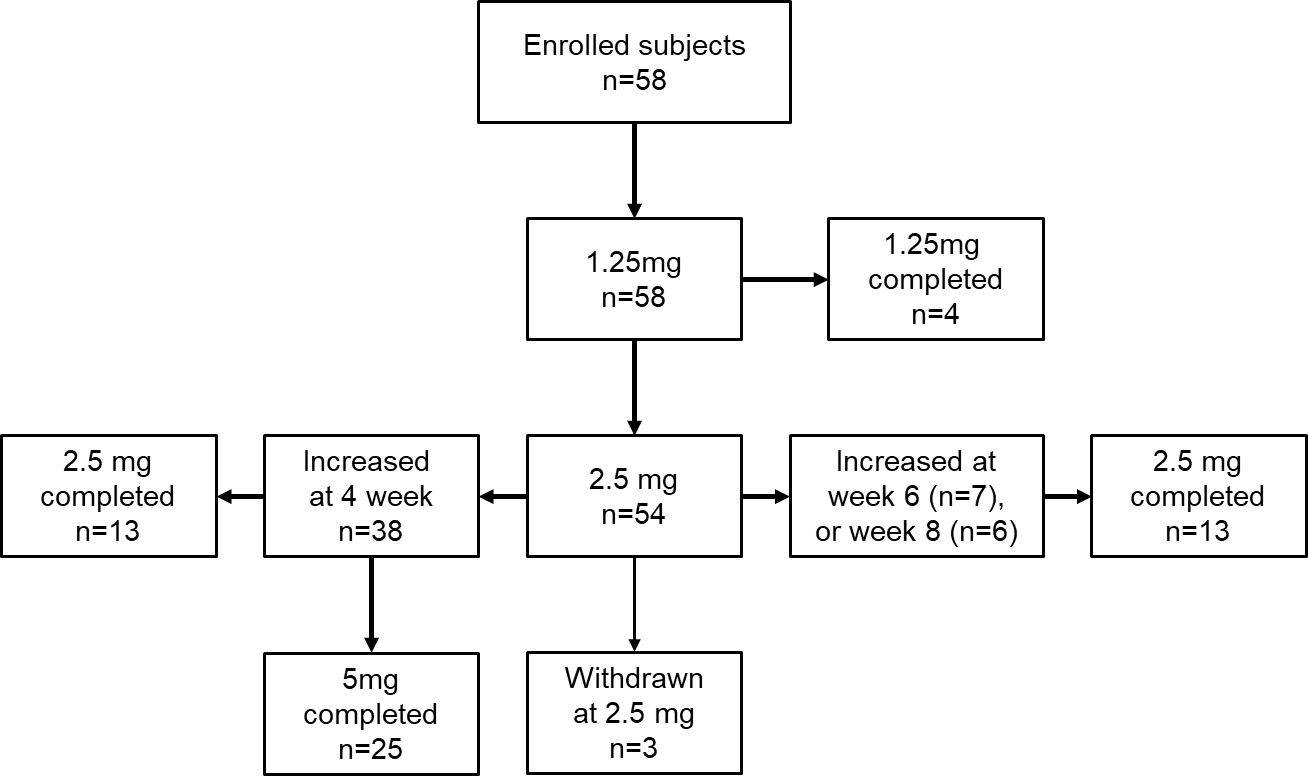
**

Dose escalation in the add-on therapy group

**Supplementary Figure 2**

**
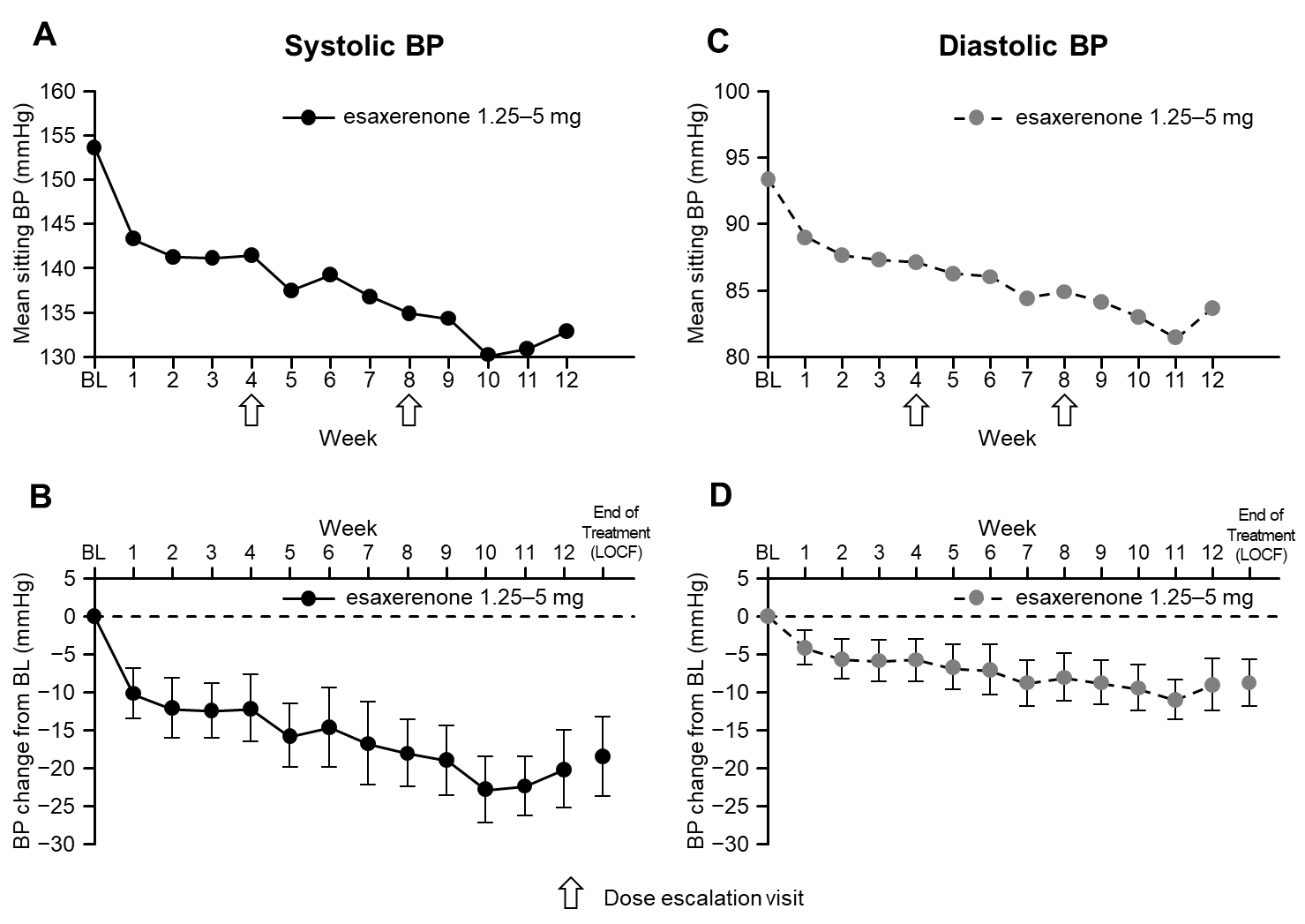
**

Mean sitting systolic blood pressure (BP) (**A**) and change from baseline (BL) (**B**), and mean sitting diastolic BP (**C**) and change from BL (**D**) in the esaxerenone monotherapy studies (full analysis set).

Values are means with 95% confidence intervals. LOCF; last observation carried forward. Arrows indicate dose escalation visits.

**Supplementary Figure 3**

**
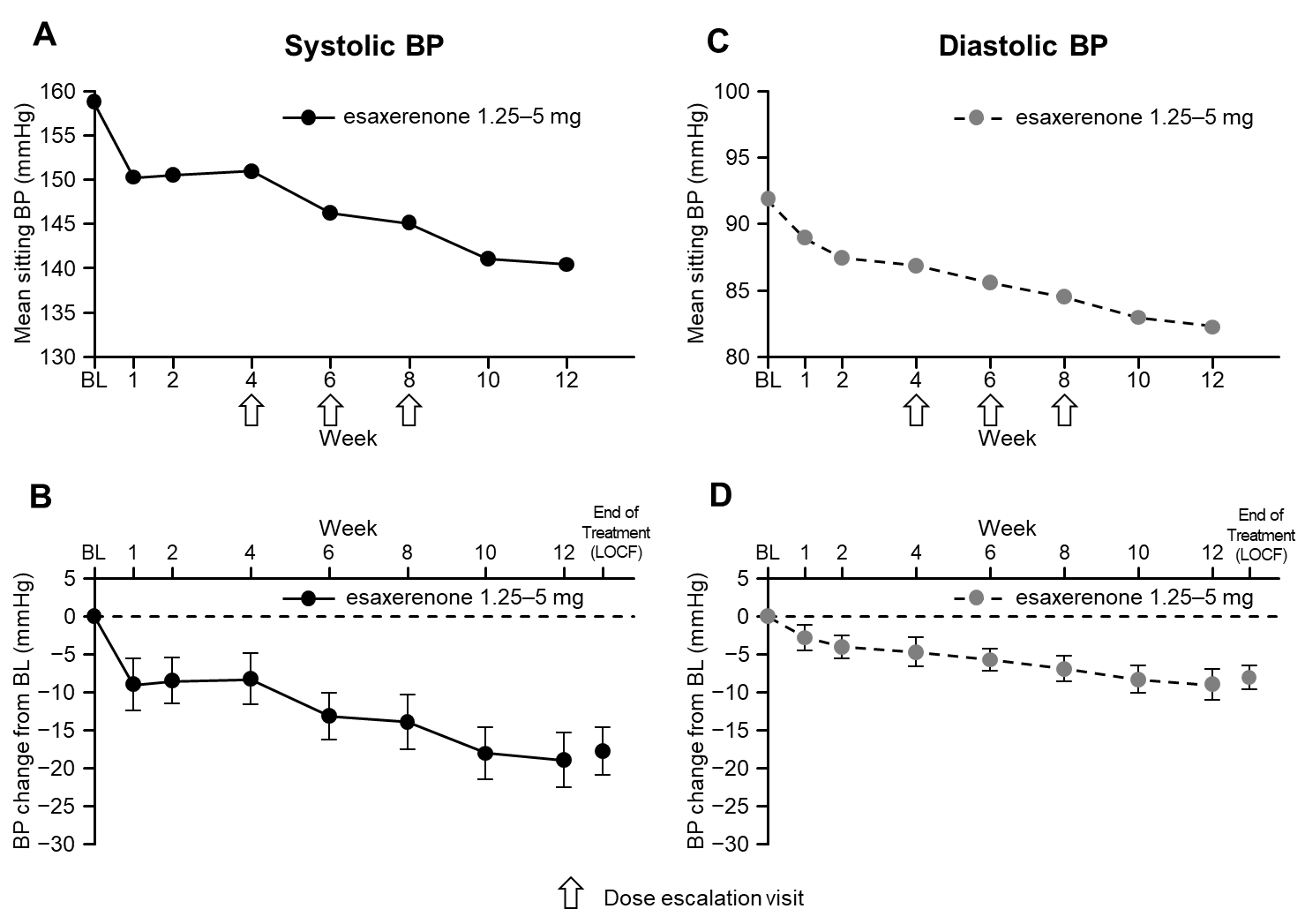
**

Mean sitting systolic blood pressure (BP) (**A**) and change from baseline (BL) (**B**), and mean sitting diastolic BP (**C**) and change from BL (**D**) in the esaxerenone add-on therapy studies (full analysis set).

Values are means with 95% confidence intervals. LOCF; last observation carried forward. Arrows indicate dose escalation visits.

**Supplementary Figure 4**

Proportion of patients achieving target blood pressure (BP) (<140/90 mmHg) at the end of the monotherapy (**A**) and add-on therapy (**B**) studies (full analysis set). Values are means and 95% confidence interval values; last observation carried forward method.

**Supplementary Figure 5**

**
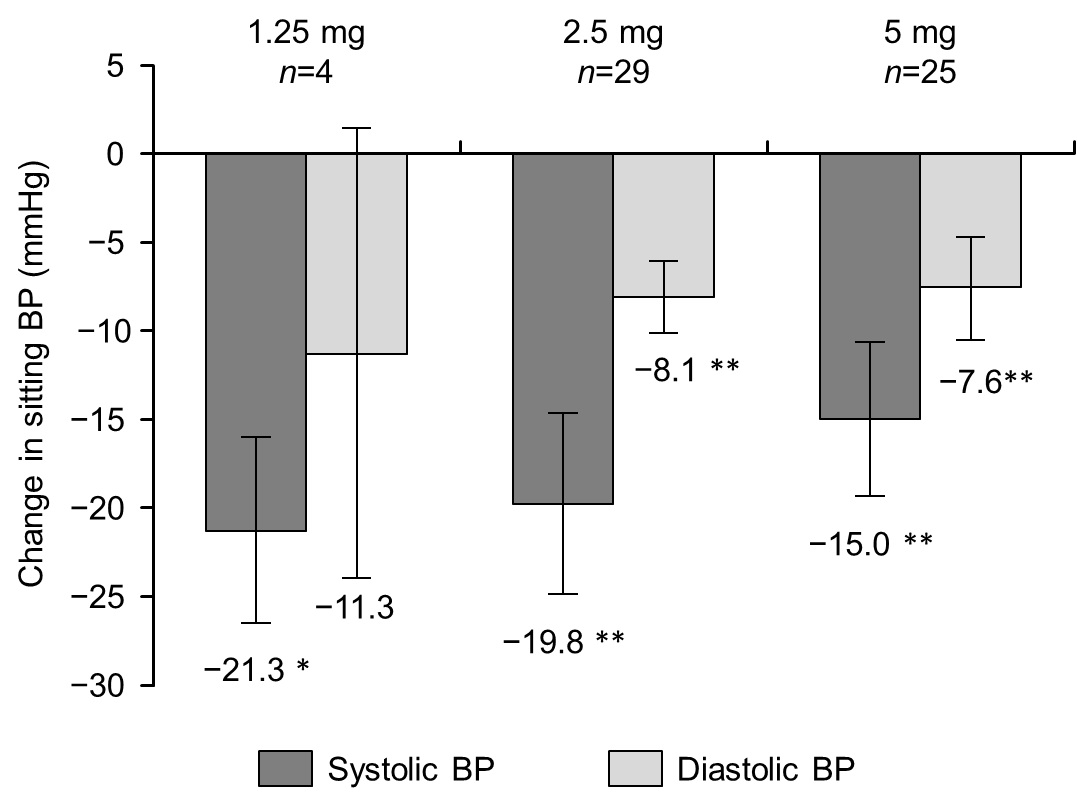
**

Mean change in sitting systolic and diastolic blood pressure (BP) in the 1.25, 2.5, and 5 mg final dose esaxerenone groups in the add-on therapy study.

Values are means with 95% confidence intervals. *P=0.001 and ^**^P<0.001 for change from baseline (paired *t*-test).

**Supplementary Figure 6**


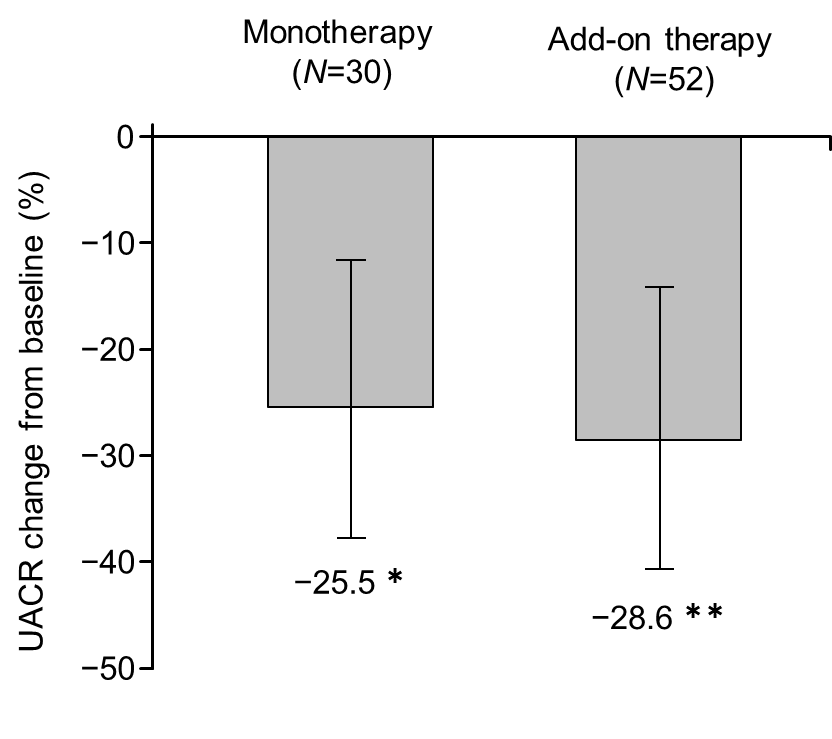


Percent change in urine albumin-to-creatinine ratio (UACR) from baseline to end of treatment for esaxerenone monotherapy and add-on therapy (full analysis set).

Values are means with 95% confidence intervals. **P*<0.01, ***P*<0.001 for change from baseline (paired *t*-test)

**Supplementary Figure 7**

**
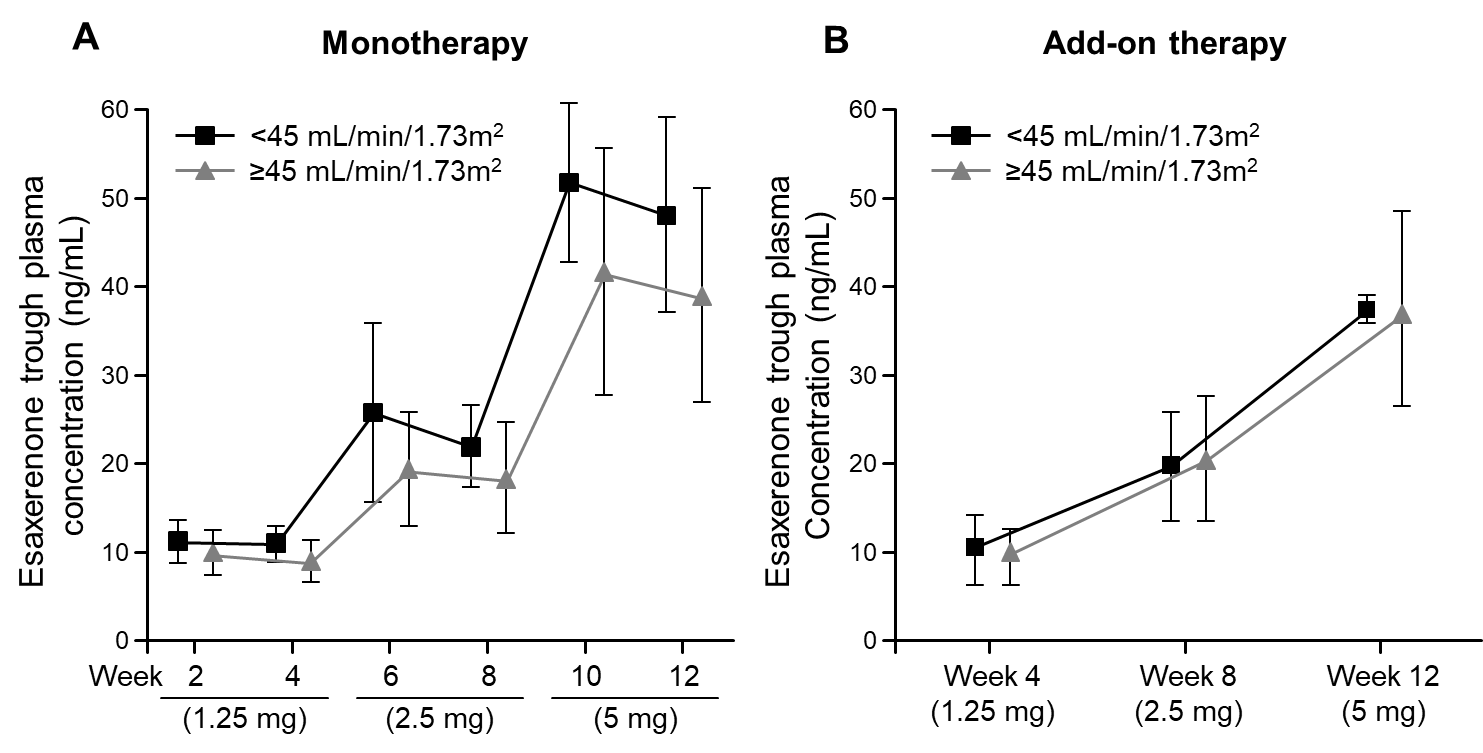
**

Trough plasma concentration of esaxerenone by baseline estimated glomerular filtration rate (eGFR; <45 vs ≥45 mL/min/1.73m^2^) in the esaxerenone monotherapy study (**A**) and add-on therapy study (**B**).

Values are means ± standard deviations.

**Supplementary Figure 8**
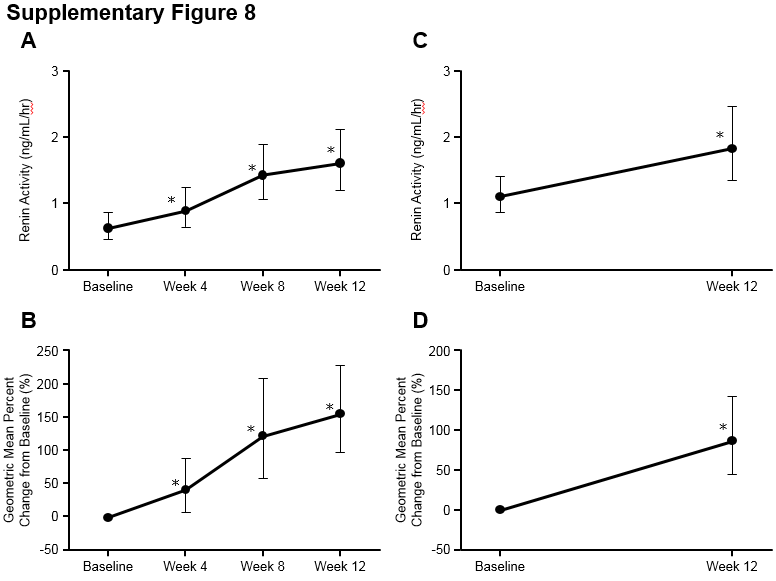


Change in plasma renin activity (**A**) and geometric mean percent change from Day 1 (**B**) in the esaxerenone monotherapy studies, and change in plasma renin activity (**C**) and geometric percent change from baseline (**D**) in the esaxerenone add-on therapy studies.

Values are means with 95% confidence intervals.

* Statistically significant difference

# Supplementary Tables

## Supplementary Table 1: Mean change in sitting blood pressure (BP) from baseline to end of treatment with esaxerenone monotherapy and add-on therapy in the full analysis set by patient subgroup

|  |  | **Monotherapy** | | | **Add-on therapy** | | |
| --- | --- | --- | --- | --- | --- | --- | --- |
|  |  | **Patients**  **(n)** | **Mean change at end of treatment,**  **mmHg (95% CI)** | | **Patients**  **(n)** | **Mean change at end of treatment ,**  **mmHg (95% CI)** | |
|  |  |  | **Systolic BP** | **Diastolic BP** |  | **Systolic BP** | **Diastolic BP** |
| Age | <65 years | 16 | −22.9  (−28.6, −17.3) | −11.5  (−15.8, −7.2) | 17 | −17.2  (−24.2, −10.3) | −8.1  (−11.2, −4.9) |
|  | ≥65 years | 17 | −14.4  (−23.1, −5.6) | −6.3  (−10.8, −1.8) | 41 | −18.1  (−21.7, −14.5) | −8.1  (−10.1, −6.1) |
| BMI | <25 kg/m^2^ | 17 | −18.4  (−25.8, −10.9) | −8.4  (−13.0, −3.7) | 29 | −19.1  (−23.7, −14.5) | −8.6  (−11.1, −6.1) |
|  | ≥25 kg/m^2^ | 16 | −18.7  (−27.0, −10.4) | −9.3  (−13.9, −4.7) | 29 | −16.6  (−21.0, −12.1) | −7.6  (−9.9, −5.4) |
| Diabetes | No | 20 | −15.6  (−23.5, −7.7) | −8.4  (−12.9, −3.9) | 45 | −17.2  (−20.9, −13.6) | −7.6  (−9.5, −5.8) |
|  | Yes | 13 | −23.0  (−28.6, −17.4) | −9.5  (−13.9, −5.0) | 13 | −19.9  (−26.7, −13.2) | −9.8  (−13.8, −5.7) |
| eGFR | <45 mL/min/1.73m^2^ | 7 | −10.0  (−28.8, 8.8) | −4.7  (−15.1, 5.7) | 12 | −19.1  (−29.2, −9.0) | −10.7  (−14.6, −6.7) |
|  | ≥45 mL/min/1.73m^2^ | 26 | −20.8  (−25.8, −15.8) | −9.9  (−13.1, −6.8) | 46 | −17.5  (−20.7, −14.3) | −7.4  (−9.3, −5.6) |

BMI, body mass index; CI, confidence interval; eGFR, estimated glomerular filtration rate.

## Supplementary Table 2: Change in urinary markers of nephropathy (add-on therapy)

|  | **Baseline** | **Week 12** | **% change** | **P value** |
| --- | --- | --- | --- | --- |
| 8-OHdG (ng/mg•Cr) | 8.0 [7.4, 8.7] | 7.9 [7.2, 8.7] | −3.3 [−12.5, 6.9] | - |
| AGT (ng/mL•Cr) | 0.09 [0.07, 0.12] | 0.08 [0.06, 0.10] | −16.4 [−30.1, −0.1] | <0.05 |
| β2-MG (µg/L•Cr) | 1.9 [1.4, 2.5] | 1.3 [1.0, 1.7] | −37.0 [−49.5, −21.3] | <0.05 |
| L-FABP (µg/g•Cr) | 2.1 [1.7, 2.6] | 2.1 [1.7, 2.4] | −4.1 [−16.7, 10.4] | - |
| NAG (IU/L•Cr) | 0.04 [0.03, 0.04] | 0.04 [0.03, 0.05] | 5.2 [−8.6, 21.1] | - |

Values are geometric means with 95% confidence intervals.

8-OHdG, 8-hydroxydeoxyguanosine; β2-MG, β2-microglobulin; ATG, angiotensinogen; Cr, creatinine; L-FABP, liver-type fatty acid binding protein; NAG, N-acetyl-β-(D)-glucosaminidase.
